# Ferruginous Pill of Mercury

**Published:** 1845-11-01

**Authors:** 


*Ferruginous Pill of Mercury.*—Prof. McLean, in the Illinois Medical Journal of June, 1845, recommends the following:

Mercury, ℥ i. Confection of Roses, ℥ iss. Sesqui-oxide of Iron, ℥ ss. Liquorice root in powder, ℥ ss.

Mix the Iron and Confection of Roses, then add the Mercury, and rub till the glob ules disappear; lastly, add the liquorice, and thoroughly incorporate it into the mass.

The object of this combination is to obtain the associated effects of the iron and mercury in cases where both are indicated; and as the former is seldom contra-indicated in such proportion as this combination embraces, it may be employed as a substitute for the Pil. Hydrarg. Prof. McLean states that "by the addition of the iron, the great labor of forming the blue pill is done away with, so that every physician ean prepare his own. I have, in 5 or 10 minutes, so reduced the mercury that no globules could be discovered by the aid of a glass magnifying from 10 to 15 times." He also states, "that a much less quantity of iron than is given above, will answer for the speedy and effectual reduction of the mercury."

Another important consideration is given in the article alluded to, viz: "Sulphuric acid is sometimes added to the conserve of roses to improve its color, which, if present, when the mercurial pill is prepared according to the usual method, forms a sub-sulphate of mercury—a compound possessing very energetic properties; but when formed with the oxide of iron, instead of this, a harmless ferruginous salt is produced, and the uniformity of the mercurial compound is not disturbed."

He sums up the advantages which this possesses over the common blue pill as follows: "1st, it is much more easily prepared. 2d, its strength is more uniform. 3d, it is more active and uniform in its operation."

